# Therapists’ experiences of remote working during the COVID-19 pandemic

**DOI:** 10.3389/fpsyg.2022.966021

**Published:** 2022-12-16

**Authors:** Andrew Morgan, Cari Davies, Yasmine Olabi, Laura Hope-Stone, Mary Gemma Cherry, Peter Fisher

**Affiliations:** ^1^Primary Care and Mental Health, University of Liverpool, Liverpool, United Kingdom; ^2^Department of Psychological Sciences, University of Liverpool, Liverpool, United Kingdom

**Keywords:** COVID-19, remote therapy, cancer, interpretative phenomenological analysis, qualitative

## Abstract

**Objectives:**

To explore the experiences of therapists who delivered remote psychological therapy during the COVID-19 pandemic.

**Design:**

This was a qualitative, phenomenological study. Interpretative Phenomenological Analysis elicited themes from semi-structured interviews.

**Methods:**

A purposive sample of eight therapists was recruited from breast cancer services in the United Kingdom.

**Results:**

Analysis identified three superordinate themes. Participants spoke about how their experience of remote working changed over time from an initial crisis response to a new status quo. They adapted to the specific practical and personal challenges of remote working and struggled to connect with clients as the use of technology fundamentally changed the experience of therapy.

**Conclusion:**

Consideration should be given to the impact of remote working on therapists and the quality of their practise. Adjustments to ways of working can help to maximize the advantages of remote working while minimizing potential issues.

## Introduction

The COVID-19 pandemic has put tremendous pressure on health care services and challenged them to work flexibly to meet patient needs (Mahase, 2021). Safety measures such as social distancing rules and concerns about cross-infection has made face to face meetings between psychological therapists and service users less common. In response, psychological therapy services began offering an increased proportion of therapy sessions remotely, *via* telephone or video call. This unusual situation represents a novel opportunity to learn more about the experience of conducting therapy remotely, during a public health crisis, from the perspective of therapists.

Psychological therapy can be defined as talking to a therapist about one’s experience and how these can influence wellbeing. Remote therapy involves meeting with a therapist *via* telephone or video rather than face to face. Remote therapy has potential benefits for service users and service providers, with many NHS psychology services offering 1–1 psychological therapy. Removing barriers to in-person attendance can help to meet the NHS’s long-standing commitment to address inequality of access to health care (Buck and Jabbal, 2014). Issues such as physical health conditions, reliance on public transport, and insufficient access to childcare disproportionately affect people from lower socio-economic status groups (Buck and Jabbal, 2014; Sakellariou and Rotarou, 2017), who are in turn more likely to experience elevated levels of psychological distress (Leung et al., 2016; Lam et al., 2019). Remote therapy also offers economic benefits to service providers by reducing the need for physical clinic space and the associated costs, as well as reducing the number of missed appointments (Vijayaraghavan et al., 2015; Shaw et al., 2017, 2018).

Despite these benefits, therapists’ attitudes toward remote working are often negative (Humer et al., 2020). Therapists can feel under-skilled due to a lack of specific training and standardized ways of working and have doubts about the efficacy of remote therapy (Bee et al., 2016; Turner et al., 2018; Knott et al., 2020; McBeath et al., 2020). Two key concerns were a lack of confidence in managing risk (Fisher et al., 2020) and feeling particularly fatigued during remote therapy (Cantone et al., 2021; Mancinelli et al., 2021).

Evidence regarding efficacy and relationship building in remote therapy is generally more positive than the perception of therapists. Intervention studies show no evidence of inferior efficacy (Watson et al., 2017; Norwood et al., 2018; Castro et al., 2020), or poorer quality of relationships (Irvine et al., 2020) when comparing remote and face to face therapy.

A recent review outlined the need for increased understanding of the breadth of factors that underpin therapists’ attitudes toward remote therapy considering this apparent incongruity (Irvine et al., 2020). This paper aims to contribute to this area by using Interpretative Phenomenological Analysis (IPA) to develop an in depth, ideographic understanding of the experience of delivering remote therapy. It also aims to explore the specific context of delivering remote therapy during a public health crisis. As an inductive method, IPA is particularly well placed for exploring a novel experience such as this (Brocki and Wearden, 2006).

## Materials and methods

### Design

This is a qualitative study which analyzed eight semi-structured interviews using IPA. Ethical approval was granted by the University Research Ethics Committee at the researchers’ institution (Ethics number: 8080).

### Sampling and recruitment

To achieve sufficient sample homogeneity for IPA, participants were purposively sampled based on their experience of delivering one to one remote psychological therapy to patients using breast cancer services during the COVID-19 pandemic. Eight participants were recruited as this number was thought to be small enough to allow sufficient depth of engagement with the data to generate rich interpretation of individual accounts, while providing sufficient breadth of experiences to allow themes to be generated across the sample (Smith et al., 2009).

Information packs outlining the study were sent to online Clinical Psychology social media groups and shared on Twitter. In addition, 40 breast cancer psychology services in the United Kingdom were approached *via* email or phone. Recruitment began in October 2020 and remained open until January 2021. Eligible therapists were invited to contact researchers to declare their interest. They were given the opportunity to ask questions about the study, and times were arranged for interviews. Due to COVID-19 restrictions on face to face meetings and geographical distance, participants were offered a choice between telephone and video call interviews: all opted for telephone. All participants read the participant information sheet and provided written consent prior to interview.

Participant characteristics are presented in Table 1. Participants were given pseudonyms to protect their anonymity and identifying information in the transcripts was removed.

**Table 1:** Participants characteristics.

Participant	Gender	Age	Ethnicity	Job title	Full time/part time	Number of years experience	Service
Alice	Female	31	White British	Macmillan psychologist	Full time	Less than a year (5 months)	Cancer psychology
Bethan	Female	63	White British	Psychotherapist	Part time	3 years in role (9 years as a therapist)	Psycho-oncology
Megan	Female	50	White British	Macmillan lead psychologist	Full time	10–12 years (18 months in psych oncology)	Cancer psychology
Sara	Female	37	White British	Clinical psychologist	Part time	5 years in psycho-oncology	Health psychology and staff support
Erin	Female	49	White British	Lead clinical psychologist	Part time	33 years (3 years psycho-oncology)	Cancer psycho-oncology team
Anna	Female	39	White British	Macmillan counselor	Part time	6 years	Clinical health psychology
Gwen	Female	41	White British	Principal clinical psychologist	Part time	13 years (8 years psycho-oncology)	Oncology
Jack	Male	40	White British	Consultant clinical psychologist	Full time	26 years (20 years in psych-oncology)	Community based physical health psychology service

All names changed to preserve anonymity.

### Data collection

Semi-structured interviews ranging from 52 to 60 min were conducted by AM. Interviews were audio recorded and transcribed verbatim. A semi-structured interview schedule (Table 2) was developed according to guidance from Smith et al. (2009). This allowed participants to discuss the topics which were most salient to them, therefore minimizing the influence of researcher presuppositions, while ensuring sufficient similarity in content to facilitate comparison between interviews.

**Table 2:** Interview schedule.

**Topic area 1: Background in remote therapy** Can you tell me about what experience you have had of delivering remote therapy? *Prompts:* How many engagements, sessions? Related to which issues? Using which models? Can you describe your physical environment?
**Topic area 2: Adjustments and decision making** Can you talk about the impact that working remotely has had on the way you conduct therapy, if at all? *Prompts:* And why/how did you make these decisions? What impact do you think this has on clients? Have your opinions/adjustments changed over time?
**Topic area 3: Experience of delivering remote therapy** Can you talk about what it has been like for you to deliver remote therapy? *Prompts:* Have you felt able to work to your normal standards of quality? Has anything been helpful or unhelpful in this regard? Have you been working from home and if so what has that experience been like? What would have been helpful to prepare you for this experience/for the future?
*General prompts:* What was that experience like? How did that make you feel?/How did you manage? Why do you think that happened? Can you talk a bit more about that please? Can you give some examples? And for you specifically? (if participant answers in general sense) Has this experience changed over time? How? How do you hope this will be in the future? Have I understood this correctly? (reflect topic back using their own words) In light of what you have just said, can we please revisit a previous question?

Open questions were used to encourage participants to lead the conversation, and prompts were used as necessary to encourage in-depth reflection (Pietkiewicz and Smith, 2014). The choice of broad topic areas was influenced by existing research and informal conversations with therapists working remotely in similar settings. The length of schedule was chosen to provide enough content for an in-depth, approximately 60 min interview where participants could answer in full detail and to their satisfaction. Following interviews, a debrief was offered to participants. Audio recordings of interviews were deleted following transcription and a key connecting pseudonyms with participants was stored securely and separately from transcriptions.

The interviews took place between 13/11/2020 and 27/01/2021. During this time the UK was in and out of national lockdown.

### Data analysis

Analysis of transcripts was informed by the work of Smith et al. (2009) and based upon an interpretivist epistemology (Gray, 2021). The analysis team (CD, an Assistant Psychologist and researcher working in psycho-oncology; AM, a Clinical Psychologist and researcher working in psycho-oncology; YO, a Trainee Clinical Psychologist) took part in an inductive, hermeneutic and iterative cycle of analysis. This involved line by line analysis of transcripts, identifying emergent themes from within individual accounts, and developing a wider structure of superordinate and subordinate themes across accounts. Instances of convergence and divergence between accounts were accounted for in the thematic structure. After the initial analytic cycle was concluded, individual accounts were re-analyzed in the context of the wider interpretative structure, ensuring a consistent connection between conceptual analysis and the initial data. At each stage of this process, analysis team meetings took place and interpretations were shared and discussed. Different potential themes were deliberated on, with the final thematic structure representing an interpretation shared by the analysis team as a whole. This process was repeated until the team agreed that they had created a legitimate, resonant interpretation of participants’ experiences which was grounded in the data.

To ensure rigor, the interpretivist foundation of IPA and the personal bias of the researchers were articulated consistently during the analytic process through reflective group discussions (De Witt and Ploeg, 2006). Researchers kept a personal reflective diary (Vicary et al., 2017), and a detailed audit trail of their analysis (Whitehead, 2004) to facilitate transparency. Members of the wider research team (PF, a Clinical Psychologist and researcher working in psycho-oncology; and LHS, a Health Psychologist and researcher working in psycho-oncology) audited a sample of analysis, including initial annotation of transcripts and final theme generation, for coherence and trustworthiness.

## Results

Three superordinate themes emerged from the data: (1) Coping with COVID: from crisis to a new way of working; (2) Is remote working real therapy?: adjusting to novel barriers in therapeutic practise; (3) Making connections through technology: the impact of technology on the therapeutic dynamic. Superordinate and subordinate themes are presented in Table 3.

**Table 3:** Super-ordinate themes and corresponding sub-ordinate themes.

Coping with COVID: From crisis to a new way of working	Is remote working real therapy?: Adjusting to novel barriers in therapeutic practice	Making connections through technology: The impact of technology on the therapeutic dynamic
An emergency response	Is this still the job I love?	The alienating effect of technology
Isolation and a sense of loss	A lack of confidence in the approach	The ineffable effect of sharing space
Adaptation over time and looking to the future with trepidation	Navigating a new landscape	The dynamics of remote therapy

### Superordinate theme 1

#### Coping with COVID: From crisis to a new way of working

All participants spoke about their experience of being faced with the upheaval of the COVID-19 pandemic and the impact, both personally and professionally. Over time they would adapt to this initial shock and find ways of providing the best service they could in trying conditions. As adjustment occurred, participants began to look forward to what a new status quo might be like.

### An emergency response

Erin described the experience of the early days of the COVID-19 response in vivid, kinetic terms as “a whirlwind” and “utter chaos.” In doing so she captured the mood of panic among participants and the sense that they were caught in something akin to a natural disaster. She articulated the common experience of feeling part of an emergency response where practical demands took priority over all else: “I did not really have time to reflect to be honest, it was just a matter of getting on with it and trying to get things up and running as quickly as possible.”

All participants experienced a great deal of uncertainty about how psychological services would continue during this time and organizing services while managing the personal impact of the pandemic was a major challenge:

It was a big unknown (…) as psychologists we don’t tend to work this way and so I really didn’t know, and the idea, the prospect of doing remote appointments, erm, felt very, very strange you know? [I] kind of had that attitude, "how would this work? You need to be in a room with the person" (…) All I knew at the time for my own, my own sanity, I needed to be at home to be able to, kind of, help manage my own stress levels to then do my job, erm, and again at the time it was very much crisis management. [Sara]

Five participants told us that a lack of support from employers meant that they were put in the difficult position of having to provide their own resources for remote therapy: proper equipment and suitable space to conduct emotionally challenging work were at a premium.^1^^,^^2^^,^^3^ This forced some participants to make tough decisions and compromises about their own emotional wellbeing and even their physical safety:

Our department didn’t provide any resources or any of that and actively said that if we didn’t feel we had the resources to work remotely from home that we would have to come into the hospital, erm, but that meant coming into an office that actually didn’t meet the socially distanced guidelines, so it was a bit of a rock and hard place decision for people. [Gwen]

### Isolation and a sense of loss

Six participants spoke about the emotional impact of being physically isolated from their place of work and their colleagues. Alice spoke melancholically about the sense of loss and disruption she experienced as a result of distancing restrictions. As with other participants, she was upset not only by how restrictions affected her personally, but also how they diminished her ability to provide her clients with the high standard of service to which she aspired:

[I’m] frustrated that I’m unable to, kind of, maybe deliver therapy in a way that I really would like to able to, I really miss the contact (…) it’s all become, yeah, remote and much more distanced (…) [I feel] sadness and loss for what I thought this year would look like and developing my skills.

I guess it’s just feeling much more isolated in the profession in terms of meetings or being able to quickly pop next door to talk to a nurse (…) or being able to do joint sessions where we can kind of bring together your, erm, skills to best help the patient. [Alice]

The loss of informal contact with colleagues was something participants also felt deeply:

We see a lot less of each other than we did before so if you have a difficult, you know, patient you know or sometimes, you know, you just have that 5 minute kettle debrief or something which, you know, can make a difference, you know? (…) It adds to your sense of isolation (…) feeling less supported because there’s less access to colleagues. [Megan]

Four participants spoke about measures they took to ameliorate the impact of isolation. Most agreed that video meetings did not replace the experience of in-person interaction with colleagues. Anna spoke with glee about her weekly, face to face coffee meetings, emphasizing that even a small amount of personal contact had a large, positive impact on her sense of isolation:

There’s one day a week where I see one of my colleagues (…) [We] grab a coffee, albeit socially distanced, and we just catch up for ten minutes or so and that actually, is really lovely [laughs] and last time I saw her we both agreed that it’s the highlight of our working week! Just to have a nice cup of coffee, which we both like, and also just say, “hello!”, face to face. [Anna]

### Adaptation over time and looking to the future with trepidation

Over time, participants began to adjust to their strange new circumstances. The initial sense of anxiety alleviated as they became more familiar with the practical and emotional demands of remote working. Bethan captured a common early fear among participants of now being ‘rubbish’ at her job:

I just thought, “I’m going to feel completely incompetent, um, and I won’t be able to do the thing I know I can do as a therapist because I’ll be so deskilled by being rubbish with the technology", but actually that didn’t materialise (…) That was my initial reaction but I have to say it was quite short lived actually. [Bethan]

Most participants commented on the importance of choice in their experience of remote working. Being forced to work from home could leave them feeling trapped with emotionally challenging material after the workday had ended. Megan spoke about how control over ones working pattern helped to mitigate this feeling. She urged caution about the importance of maintaining healthy work-life boundaries when looking to a post-pandemic future:

[If] you’re making an active choice to, you know, work from home because, erm, that serves your purpose then that’s fine. If you’re having to do that because you have no choice, then I think, then your home becomes work then doesn’t it? And so then you don’t then have that separation and it's much more difficult to, to kind of look after yourself and not take things home cos you literally are doing those things at home. [Megan]

Despite a degree of trepidation, most participants spoke with a sense of optimism about the positive impact the newfound flexibility of remote working could have for both themselves and their clients. Alice told us how these changes to her role had been unexpected, but ultimately welcome. She was enthusiastic about the freedom afforded to her by remote working and the impact this had on her quality of life:

The flexibility that it gives us as psychologist to be able to work from home, I never thought I’d see the day! But you know, it does introduce a bit more flexibility to what our role can look like (…) For some patients they really like having the option to have a remote session and especially if they’re working or have, sort of, childcare commitments (…) Having one day a week where you can work from home just allows a bit more flexibility (...) I’ve been pretty grateful for that actually and probably its allowing me to do a bit more of things without having the commute that day, and yeah, just get up and do some yoga [laughs]. [Alice]

### Superordinate theme 2

#### Is remote working real therapy?: Adjusting to novel barriers in therapeutic practise

All participants spoke about difficulty in adapting their usual ways of working to the novel circumstances of remote therapy. This change represented a challenge to their professional identities, their perception of how they as a therapist *should* deliver therapy, as well as their confidence in their abilities and in the techniques and approaches they had previously relied on. Participants’ responses to this challenge varied as, faced with a lack of previous experience or professional guidance, they drew on their personal values and resourcefulness.

### Is this still the job I love?

Six participants spoke about the negative impact remote working had on their job satisfaction and how they tried to meet this challenge with a sense of pragmatism:

I don’t find it quite as satisfying (…) I found that a bit, umm, demotivating at times and it's been harder to, normally I’d go off to work with a spring in my step and I really enjoy what I do and its, just felt undefinably unsatisfying (…) It's become clear that that’s something that’s here for a long time rather than just a short term response to a crisis, that’s affected my motivation and made me feel a bit a bit flat and lacking in energy about it sometimes.

The pragmatic side of me is trying to just be at peace with, at the moment, this is the best we can offer. [Bethan]

Despite this dissatisfaction, these participants took heart from the fact that they were doing their best in a difficult situation, and in doing so identified their professional role as one of helping people using whatever means available. Jack spoke in decisive terms about how the concepts of duty and flexibility were central to his identity as a psychologist:

Not offering a service is not an option (…) [You] owe it to, you know, a patient and you owe it to your other colleagues in the NHS to do your bit (…) You apply the basic principles, you think, “well if this person is prepared to talk to me by video, or more usually phone, umm, I should be prepared to talk to them and we’ll work it out together.” [Jack]

Two participants described their experience of remote working as an unsatisfactory substitute for face to face therapy. The separation they felt from clients diminished the quality of the therapy and this was detrimental to their sense of professional identity, leaving them feeling that the nature of their role had fundamentally changed. Megan said, ‘I feel like I work in a call center these days’, describing the work as ‘soul destroying’ and questioning if what she was offering was ‘really psychological therapy’ or something else entirely.

Gwen felt similarly and spoke passionately about her discontent:

I absolutely hate it! To be absolutely frank on that, I hate it (…) I feel a real disconnect with patients that I’m seeing using this modality. I don’t feel like I know people in the same way.

In terms of me and what I’m happy with and what I know I can offer, I don’t, I don’t feel like patients are getting the same quality of input. [Gwen]

She concluded, ‘If that’s going to be the case this will not be a job that I’ll want to do’.

### A lack of confidence in the approach

The unfamiliarity of remote working led participants to avoid certain exercises, techniques or interventions which they felt could otherwise have been helpful for their clients. Alice was typical in feeling anxious about managing clients’ distress when working remotely:

I’ve struggled with doing more experiential exercises so things like, ermm, behavioural experiments or (…) breathing exercises in, sort of, panic, more panic focused interventions, and I have had that with a few breast cancer patients where it's very difficult to create the safety, ermm, needed to do those experiments (…) I’ve probably found myself maybe avoiding them more or not doing the exercise that I think probably are what might create the change for people, ermm, so that’s yeah that’s been frustrating. I have found actually with a few patients that they have become very distressed during the session over video and it’s just been really difficult to manage that … in ways that normally I felt would have worked, ermm, more effectively. [Alice]

Most participants spoke about feeling as though some of their therapeutic tools had been compromised by working remotely and expressed concern about how this might impact on the quality of their work. Gwen felt that she had lost a fundamental part of her skillset as a therapist:

I’m missing the use of the rest of my body, that I would say that in my practice I’m probably quite a physical clinician in a way that I’ll demonstrate quite a lot of things. So if I’m kind of looking at a particular ACT concept with somebody I might [get] people to hold the folder and pushing against it then you know seeing what it feels like to sit with that resistance and I think these things really stick with people you know? [Gwen]

Technical issues were another reason for anxiety among participants and were an obstacle to providing the quality of service they aspired to. Sara was emphatic in her language and tone when describing the anxiety that accompanied the unpredictable performance of the technology upon which she was now wholly reliant. She captured the sense of panic and frustration participants felt when factors outside their control contributed to the distress of clients and reduced the quality of their work:

[Its] very stressful, very, very stressful, again when you think about wanting to provide that containing safe and secure environment for somebody. If you’ve got somebody glitching, or maybe you think that you’re glitching, it it just doesn’t feel you can give that you know that containment or have that connection with somebody. (…) so that is very, very difficult. [Sara]

Regardless of their therapeutic orientation or sense of professional identity, participants were unanimous in their agreement that remote working was not as good as face to face. The following statement from Jack was particularly resonant given his unequivocal defense of a pragmatic professional identity. Although he remained fully committed to this, he was clear about his belief in the superiority of face to face working:

In the end of the day, umm, we all know even a phone call or a video link is a denuded form of conversation, it’s not the same as being in the room with someone, it’s not gold standard. [Jack]

### Navigating a new landscape

Participants’ attempts to meet the new challenges of remote working led to significant changes in how they delivered therapy. They found themselves drawing on their personal ingenuity and adaptability in light of a lack of established guidance or training. One of the major obstacles participants faced was a loss of visual information, particularly during telephone work:

[I’m] having to think and maybe adapt a bit too especially when it’s over the phone. So more verbal acknowledgement of the difficulty they’re experiencing and lot of normalisation, ermm, but yeah I think I really feel the loss of being able to show that in your own expression of body language and, ermm, that's something of the real downside to, to working remotely for me. [Alice]

Sara was representative of participants in talking about the feeling of a loss of the collaborative aspects of face to face therapy. Like others, her desire to provide the most helpful service possible led to her making adjustments to her approach. She did this by directing more of her efforts to work outside of therapy sessions:

[Previously] I might have started to write something and then maybe encourage somebody, you know, handed the paper over (...) like you're making a list of something with somebody you might do that together (…) I kind of used that approach slightly less which is a bit of a shame really because people, people have perhaps less to take away (...) I'll probably put more effort into [the assessment letter] because that, and the ending letter as well, because that's almost the, the thing when they don't have these other things. [Sara]

Half of participants reflected on the advantages of remote working. Jack gave the example of the additional information that he could gather about his clients, particularly during video sessions. He felt that seeing a client in their personal context could help him learn about them and form a therapeutic relationship:

You do potentially get to see the inside of people’s houses, umm, so that gives you extra clues (…) you’re looking to see what have they got on their walls you know, what are their interests? [Jack]

### Superordinate theme 3

#### Making connections through technology: The impact of technology on the therapeutic dynamic

All participants identified a personal connection with their clients as being a key aspect of high quality therapeutic work. They described how the use of technology had a profound impact on this connection and how its influence on the therapeutic dynamic fundamentally changed their experience of therapy.

### The alienating effect of technology

Over half of participants spoke about their sense of technology acting like a barrier and impeding their ability to feel fully immersed in the experience of therapy. Particularly with regards to video, participants described the impression that they were watching therapy take place with a peculiar sense of distance. Bethan contrasted this sense of witnessing therapy from the 3rd person perspective with being truly present in the moment, alongside the client in person. She felt this was detrimental to her personally and to the therapy as a whole:

In a face to face situation I could just sit with someone and, just the presence, umm, they understand that you’re sharing or empathising or witnessing their distress, but there’s something about witnessing it on a screen that’s profoundly uncomfortable for me and makes me wonder what its like for the patient, umm, because it can almost feel voyeuristic in that moment. It’s a very odd feeling. [Bethan]

Participants described how they felt they had to struggle to overcome the obstacle of technology, and despite their exertions found themselves unable to replace the immediacy and immersion of face to face therapy. Erin provided an evocative metaphorical comparison between the lack of depth of a two-dimensional image and relating to another human being through the lens of technology:

It feels always a bit like swimming in water so that everything's a little bit clouded (…) the screen feels like a tangible barrier that you're always having to work hard to overcome (…) You're always having to strain a little bit to make sure you really do hear what they're saying and to look really hard at them to pick up everything that you can feel and even when someone is, that's a really good connection and I don't, I don't think it's ever crystal clear. Somehow there's still the loss of depth.

You see a smaller snapshot of people, ermm, a flat, you get a flatter view of them (…) there is a missing dimension. [Erin]

### The ineffable effect of sharing space

All participants spoke about the powerful impact sharing the same physical space had on facilitating connections with their clients. All participants struggled to articulate this experience, which is particularly striking given the many years of experience they have in reflective practise as a core competency of the psychological therapy professions. Anna was representative of several participants in calling on abstract concepts like ‘energy’ to capture her felt sense of connection, and defining the experience of ‘being’ with a client as involving a resonant, personal encounter, sharing a physical space and time:

I don’t know if I can put it into words, but I think there’s just something about the energy I guess of being in a room with someone and being, person right there, in that space, in that time with that person. [Anna]

Gwen contemplated the impact of rituals on their experience of therapeutic connection. For her, connection was partly a metaphysical, emergent property of the communal behaviors that therapists and their clients engaged in, in their special space. She was clear that, as far as her experience was concerned, this was a ‘real’ thing that defied straight forward, empirical definition or explanation:

I think it starts before you’re even in the room. I think there’s something about going out to a waiting room and calling for somebody you know and shaking their hand (...) and accompanying that person through into the room together (...) and when you close the door you’re kind of creating a little box in a way (…) All those sort of rituals that are part of it and also just setting the scene (…) to make sure that somebody feels like cared for (…) it feels to me like that’s part of feeling that the person’s there and that they’re real (…) I don’t know if I can actually give any more specific detail as to why I feel that sense of disconnect, I just know that I do. [Gwen]

Despite the ineffable nature of this experience, participants agreed that sharing space made it “easier to feel a kind of connection… it feels more, kind of, human” [Megan].

### The dynamics of remote therapy

Five participants spoke about how, as technology makes therapy more accessible, it also requires less commitment from clients and this had an impact on the interpersonal dynamics of therapy. Less commitment led to less of a sense of collaboration and left participants frustrated, feeling as if they were directing sessions more than they would have previously. They expressed concerns that this meant not empowering clients to find their own solutions or take responsibility for their wellbeing. For Megan the barrier of entry of attending therapy in person also represented an investment for clients, leading them to be more actively engaged in the process:

[Its more difficult to] have a therapy session with them rather than it just becoming just kind of information giving session (…) I think they’re less, they’ve had to do less to be present in the session, so actually are they consenting to [therapy] or what do they think they’re consenting to? Which actually, by getting on a bus and coming to a hospital and sitting in the session involves them making an active commitment for that process which, ermm, kind of impacts the general dynamic I think of appointments but particularly for psychology.

Patients don’t remember that you’re supposed to call them, ermm, and so (…) they’ll be on the bus or their in the bath, I’ve had quite a few people in the bath during my sessions! (…) When patients come to a hospital appointment they, kind of, prepare themselves for what the appointment is about and why, and they, they make a bit of a commitment and investment to it. [Megan]

Three participants voiced concerns about their own ability to be fully committed to therapy when working remotely. Erin spoke with a sense of guilt and self-reproach about times when she failed to be emotionally present during remote work as a direct consequence of the real and metaphorical distance between her and her clients:

Sometimes when you're sitting on the phone and you know that you haven’t got the visible presence of someone (…) I have on the very, very odd occasion checked an email in the middle of a session which is awful, and I hate to admit that, but I guess there is a possibility for that when someone can't see you erm and, that’s appalling really isn’t it? (…) When you're virtual it’s easier to slightly zone out a bit I suppose and there's something that's less present about everything. [Erin]

Despite these additional challenges, some participants were also keen to talk about how the additional emotional distance created by remote working had sometimes had a positive effect on their experience of therapeutic dynamics:

A couple of young people who weren’t really engaging well when I’d seen them face to face seemed to engage much better remotely through video appointments (…) She found it less confrontational that, because she didn’t have to come to a big hospital and sit in a room with, you know, a professional person. That she was in the comfort of her own home and, ermm, kind of felt like a sense of slight removal from the situation that she was able to share more. [Gwen]

Gwen was enthusiastic about the idea that remote therapy offers more flexibility for clients and therapists and can therefore ultimately be a positive thing if other issues can be attended to.

## Discussion

This study sought to develop an understanding of the experience of therapists delivering remote therapy within the specific context of the COVID-19 pandemic. Participants’ experiences coalesced into three themes: (1) Coping with COVID: from crisis to a new way of working, (2) Is remote working real therapy?: adjusting to novel barriers in therapeutic practise and (3) Making connections through technology: the impact of technology on the therapeutic dynamic.

All participants spoke about how technology acted as a barrier to communication and the loss of visual information, especially during telephone therapy, was identified as one key factor. They felt disarmed without the direct feedback from facial expressions of clients and less able to communicate empathy through their own non-verbal cues. This experience is consistent with previous research on remote therapy (Fisher et al., 2020; James et al., 2022) and remote communication in general: more visual information means more options for the “expression and reception of affiliate cues and the effortless processing of these cues” (Sadikaj and Moskowitz, 2018).

The more surprising insight from this current study is that video therapy was sometimes experienced as even more alienating and unnatural than telephone therapy, despite the presence of visual cues. This experience was particularly hard for participants to articulate and explain. Approaches to psychology which do not rely on introspection such as Evolutionary psychology, and specifically Media Compensation Theory (MCT) (Hantula et al., 2011), are well suited to help explain this counter intuitive finding.

As a product of evolutionary adaptation, social communication functions optimally within a particular set of situational parameters. These include the communicator’s expectation of certain informational cues. The “cue removal principle” suggests that, where expected cues are removed, communication is experienced as more effortful, unnatural and disorientating than communication where those same cues are absent, but where this is in line with expectations.

In the case of remote therapy, speaking on a telephone is similar in functional terms to talking to someone who is out of your line of sight, which has been part of the environment of evolutionary adaptation. We do not expect to be able to see someone’s facial expressions or read their body language and so our mental focus can be singular and disorientation is minimized.

Conversely, seeing someone on a video screen without some of the additional cues which we expect from face to face communication such as body language and smell, can be experienced as alienating and ‘unnatural’, as was the case for participants. It is not just the presence of additional information in the form of visual cues which is relevant to the experience of delivering remote therapy, but also the absence of expected information.

Construal Level Theory (CLT), a theory of social psychology, is another approach which can help to explain some fundamental aspects of the experience of remote therapy which may be outside of conscious awareness. CLT proposes that different types of psychological distance (e.g., space or time) are interrelated in that they represent different ways in which the object of interest is separated from the central reference point of the self. Psychological distance of any sort is circumvented by mental abstraction: the more distant an object, the more abstract it becomes and the more removed from direct experience (Trope and Liberman, 2010; for a review of supporting research see Trope and Liberman (2010). Construal-level theory of psychological distance).

This theory can help to explain why participants found making emotional connections with physically distant clients to be particularly challenging. The psychological distance entailed by being in different rooms necessitates mental abstraction of the client, which then impacts on other aspects of the relationship such as the ability to feel emotionally connected with them in the ‘here and now’. This common goal of participants, and therapists more generally, is in some sense fundamentally opposed to the abstraction required to engage with a physically, and therefore psychologically remote client.

The additional mental demands entailed by the lack of visual information, the removal of expected cues and the need for abstraction also help to explain why participants, and other research studies (Cantone et al., 2021; Mancinelli et al., 2021), found remote therapy even more effortful than face to face therapy.

Participants’ accounts show that, while technology is able to circumvent barriers to engagement, it can also act as a barrier itself. Increasing therapists’ awareness of these issues can help to manage expectations and minimize the self-criticism that participants engaged in as a result of feeling unable to meet their usual standards of making connections, feeling grounded, stamina *et cetera*. It should also be expected that clients will experience the same issues and therefore a shared awareness and allowance for this should be part of the initial set up of therapy. Awareness of these evolutionary and social mechanisms can help to explain the gap between therapist’s pessimistic outlook on remote working and the more encouraging evidence of its efficacy (Irvine et al., 2020), and specific measures such as specific additional focus on grounding of therapist and client can help to remediate it.

Perhaps the most divisive issue in this study was the impact of remote working on professional identity. A model of social work theory, ‘Person-environment fit’, explains that working in a given environment will feel more or less satisfying depending on the personal values of the professional (Carpenter and Platt, 1997). This model therefore predicts variation within groups of professionals, as was the case with participants; six found they were able to reconcile their professional identity with remote working, while Megan and Gwen were not.

The ones who were able to achieve reconciliation identified primarily with the caring elements of their profession. They had a sense that a good professional is one who does their best to help with whatever means available, and therapy is a part of this. This set of values was flexible enough to be satisfactorily expressed even within the context of remote working and crisis response. Megan and Gwen saw being an excellent therapist as the core of their professional identity. When the environment made meeting their personal standards impossible, they experienced intense dissatisfaction and considered leaving the profession. The development of professional identity is typically a long-term process which occurs over years of training, supervision and experience (Alves and Gazzola, 2011). At the time of interviews, participants were reeling from the shock of their role changing effectively overnight.

High attrition rates of health care staff continue to be a major issue in the NHS (Palmer and Rolewicz, 2022). In order to promote job satisfaction and staff retention, it is important that organizations are flexible and create environments which allow professionals with different values to work in ways which are consistent with their professional identities. Participants were clear that personal choice of which modalities they used was crucial. In the latest annual NHS staff survey (NHS, 2022) just over half of respondents felt they had choice and were involved in decision making related to their work, down from the previous year. Increasing choice and involvement in decision making should therefore be a priority for organizations.

### Implications for practice

Participants spoke about advantages of remote working which they hoped would be maintained after the crisis response was over. They valued highly the flexibility afforded to them by remote working. Less pressure on room space meant that they had more choice in terms of which clients they saw and when. Less commuting time and being able to work from home more often meant a more positive work-life balance. NHS staff survey respondents agree that flexible and remote working have been two of the main areas of their roles which have worked well since the COVID-19 pandemic and want them to be continued (NHS, 2022). Focusing on supporting staff wellbeing through facilitating flexible and remote working is also consistent with the NHS’s Long Term Plan (NHS, 2019), two of the core points of which are ‘Doing things differently’ and ‘Backing our workforce’.

Participants speculated that for clients too, the ability to engage in therapy from home could be beneficial for various practical and clinical reasons, for example not having to continually return to a place of trauma such as a hospital, or take as much time off work, improving access to those who are palliative (Moscelli et al., 2018; Guzman et al., 2020). Participants also spoke about some disadvantages including concerns about a potential lack of engagement from clients due to reduced commitment required to attend sessions (Stefan et al., 2021). Other issues include a lack of confidentiality at home for clients and maintaining an agenda of avoidance for clients with anxiety issues (Boldrini et al., 2020).

Given that remote modalities have their own distinct advantages and disadvantages, the decision of which modality should be offered to clients should be based on collaborative, person centered formulation (Mind, 2021) and revisited during the engagement. Therapists should be given some choice as part of their job plan as to how they meet the need for different therapy options. An online survey of 335 therapists also found this to be a popular demand (McBeath et al., 2020) and it is consistent with the findings of a recent mixed methods study of remote therapy during the pandemic (James et al., 2022).

Participants also asked for additional, remote-specific therapy training, the same therapist survey as above also found this to be a popular demand (McBeath et al., 2020). As well as core skills training, it is likely that specific therapy models will adapt to meet the specific demands of remote working. At the time of interviews EMDR was not yet widely considered to be suitable for remote working but this has changed due to subsequent research and practise (Tarquinio et al., 2021). Training will not only raise therapist confidence but teach useful, remote-specific skills.

Participants appreciated opportunities for reflective practise and peer support in the workplace to help mitigate isolation and allow them to process their own experiences (Billings et al., 2021). They were adamant that some degree of face to face contact with colleagues was vital. These opportunities should be provided as part of good practise at any health service which employs a mixed model of remote and face to face contact.

### Limitations

This study has a potential self-selection bias due to using a volunteer sample. Although participants offered a range of experience and perspectives, only a small proportion of people who heard about the study offered to take part. Interviews and analysis capture a specific experience which is intrinsically connected to a particular time point of the COVID-19 pandemic. Participants spoke about how their experience of delivering remote therapy changed over time due to various factors and therefore it is reasonable to assume that this change would continue as their personal and social context shifted around them. Our single-interview design cannot capture the depth and breadth of longitudinal changes. An alternate design employing multiple interviews could potentially shed some light on this aspect. Given this is an IPA study this experience is irreducible from this context and generalizations should be made cautiously and with this in mind.

### Future research

Participants wondered what the experience of remote therapy would be like for clients. A similar study design should be repeated with therapy clients with experience of both remote and face to face therapy. All participants in this study worked in breast cancer psychology services. This means that most clients had experience of this one specific physical health condition and were presumably predominantly female. Future research could explore the experiences of therapists working with different client groups. Given the meaningful differences between remote modalities, an in-depth comparison of the experience of video and telephone therapy could be insightful. Although this study chose to focus on professional identity, given the ideographic emphasis of IPA there are likely to be meaningful interactions between other aspects of identity and experiences of remote working. Future studies could consider other approaches to identity.

## Conclusion

This study provides an in depth exploration of the experience of delivering remote therapy during the COVID-19 pandemic. A range of challenges were identified by participants including working with the barrier of technology, having to come to terms with delivering what they at times perceived to be a lower quality of service and feeling isolated from their peers. Participants demonstrated flexibility and resolve in overcoming these challenges and spoke with a sense of optimism about future ways of working which harnessed the advantages of remote therapy. It is incumbent on organizations to create professional environments which realize these advances, through providing training, resources and flexible job plans, while minimizing the impact of disadvantages identified by participants.

## Data availability statement

The raw data supporting the conclusions of this article will be made available by the authors, without undue reservation.

## Ethics statement

This study involved human participants and was reviewed by the University of Liverpool Ethics Committee. The participants provided their written informed consent to participate in this study.

## Author contributions

AM, CD, and YO were responsible for the recruitment, qualitative analysis and write up. LH-S and PF were responsible for the analysis audit. All authors contributed to the conception and design of the research study, manuscript revision, read, and approved the submitted version.

## Funding

Funding is made available by the University of Liverpool for Open Access publication.

## Conflict of interest

The authors declare that the research was conducted in the absence of any commercial or financial relationships that could be construed as a potential conflict of interest.

## Publisher’s note

All claims expressed in this article are solely those of the authors and do not necessarily represent those of their affiliated organizations, or those of the publisher, the editors and the reviewers. Any product that may be evaluated in this article, or claim that may be made by its manufacturer, is not guaranteed or endorsed by the publisher.

## Footnotes

^1^(…)indicates words have been omitted to enhance fluency.

^2^[ ] indicates where words have been replaced to provide additional context.

^3^A line break indicates a separate quote from the same participant occurring elsewhere in the transcript.
